# Colonic mucosa–associated lymphoid tissue lymphoma: A case report

**DOI:** 10.3389/fsurg.2023.1178394

**Published:** 2023-04-26

**Authors:** Dan Chen, Ding-Fu Zhong, Yi Yang, Si-Shuang Chen, Dong Liu

**Affiliations:** ^1^ Department of Gastroenterology, Affiliated Jinhua Hospital of Wenzhou Medical University, Jinhua People's Hospital, Jinhua, China; ^2^ Department of Pathology, Affiliated Jinhua Hospital of Wenzhou Medical University, Jinhua People's Hospital, Jinhua, China; ^3^ Department of Hepatobiliary and Pancreatic Gastroenterology, Affiliated Jinhua Hospital of Wenzhou Medical University, Jinhua People's Hospital, Jinhua, China

**Keywords:** colonic mucosa–associated lymphoid tissue lymphoma, electronic staining endoscopy, pigmented endoscopy, magnifying endoscopy, treatment

## Abstract

**Background:**

Mucosa-associated lymphoid tissue (MALT) lymphoma is a group of extranodal lymphomas that originate from B cells. Primary colonic MALT lymphoma is a rare disease, and there is no consensus on its endoscopic features and standard therapies. It is essential to raise awareness of colonic MALT lymphoma and choose the appropriate treatment.

**Case presentation:**

In this case report, we describe a 0-IIb-type lesion that was found by electronic staining endoscopy and magnifying endoscopy. The patient underwent a definitive diagnostic ESD for diagnosis. The patient was evaluated for lymphoma after diagnostic ESD according to the Lugano 2014 evaluation criteria, which are divided into imaging remission on the basis of CT and/or magnetic resonance imaging (MRI) evaluation and metabolic remission on the basis of PET-CT evaluation. Based on the PET-CT results suggesting increased glucose metabolism in the sigmoid colon, the patient underwent additional surgical treatment. According to the pathological results of the surgery, we found that ESD could treat such lesions, which may provide a new option for colorectal MALT lymphoma.

**Conclusion:**

The low incidence of colorectal MALT lymphoma, especially for 0-IIb lesions, which are difficult to detect, requires the use of electronic staining endoscopy to improve the detection rate. The combination with magnification endoscopy can improve the understanding of colorectal MALT lymphoma, which ultimately requires pathological support for diagnosis. According to our experience with the present patient case, ESD seems to be a feasible and economical choice for the treatment of massive colorectal MALT lymphoma. However, the combined application of ESD and another therapy scheme needs further clinical investigation.

## Introduction

Mucosa-associated lymphoid tissue (MALT) lymphoma is a subtype of non-Hodgkin's lymphoma derived from B cells in the peripheral follicular area of lymph nodes. Colorectal MALT lymphomas account for only 1.6% of all MALT lymphomas, which suggests a low incidence of colorectal MALT lymphoma. Therefore, the clinical characteristics, especially the endoscopic features, and standard therapy of colorectal MALT lymphoma have not been clearly established. In this case report, we describe a 0-IIb-type lesion that was found by electronic staining endoscopy and magnifying endoscopy. The patient underwent a definitive diagnostic ESD for diagnosis. According to the primary gastrointestinal lymphoma Lugano staging system (1), the case was classified as IE1 stage. The patient was evaluated for lymphoma after diagnostic ESD according to the Lugano 2014 evaluation criteria (2), which are divided into imaging remission on the basis of CT and/or magnetic resonance imaging (MRI) evaluation and metabolic remission on the basis of PET-CT evaluation. Based on the PET-CT results suggesting increased glucose metabolism in the sigmoid colon, the patient underwent additional surgical treatment. According to the pathological results of the surgery, we found that ESD could treat such lesions, which may provide a new option for colorectal MALT lymphoma.

## Case presentation

**Figure 1:**
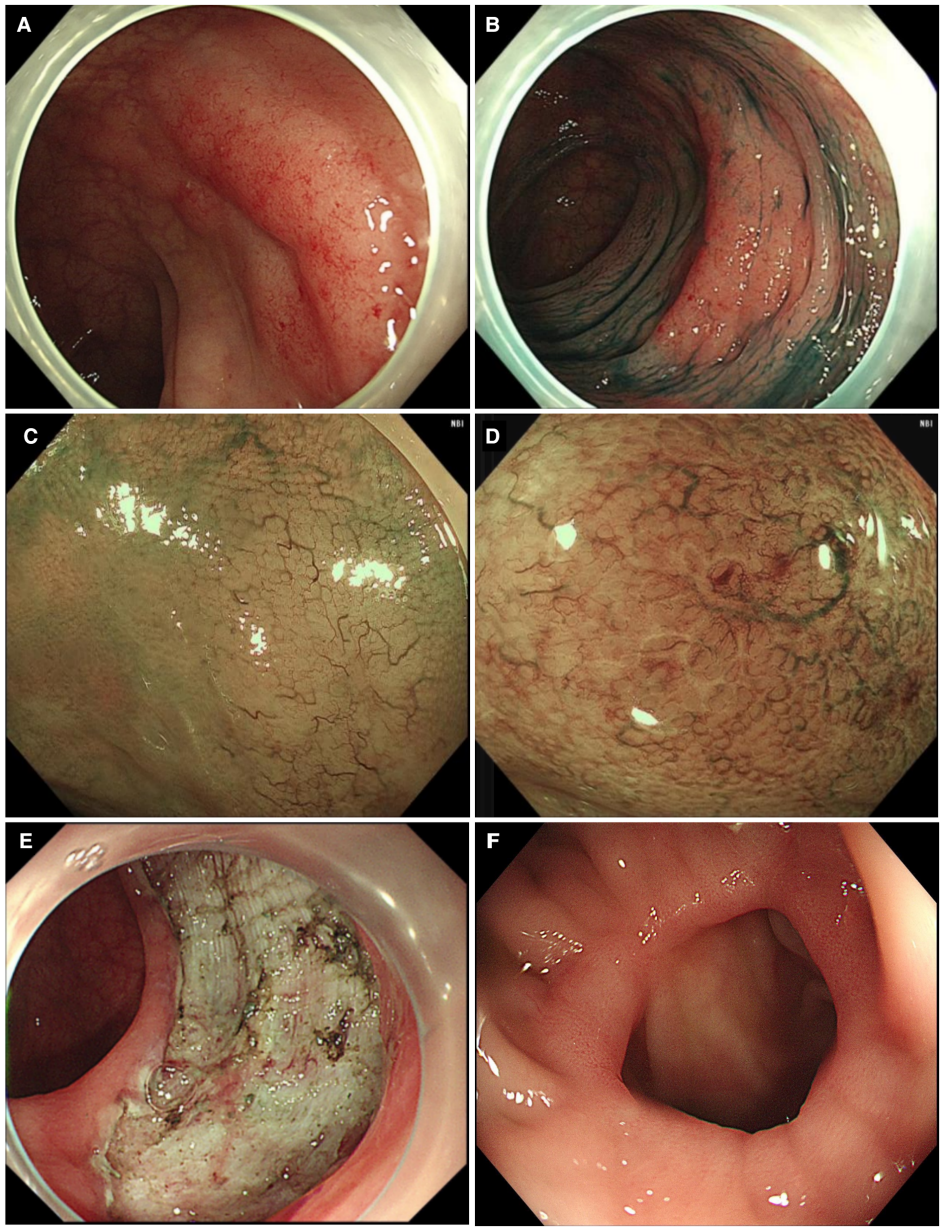
Clinical data of the patient with colonic mucosa–associated lymphoid tissue lymphoma. (**A**) Normal colonoscopy shows a 0-IIb-type lesion; (**B**) indigo carmine staining; (**C**) narrow-band imaging magnifying endoscopy shows an irregular branching vasculature; (**D**) disappeared glandular structure; (**E**) diagnostic ESD resection of lesions; (**F**) colonoscopy results after the operation.

**Figure 2:**
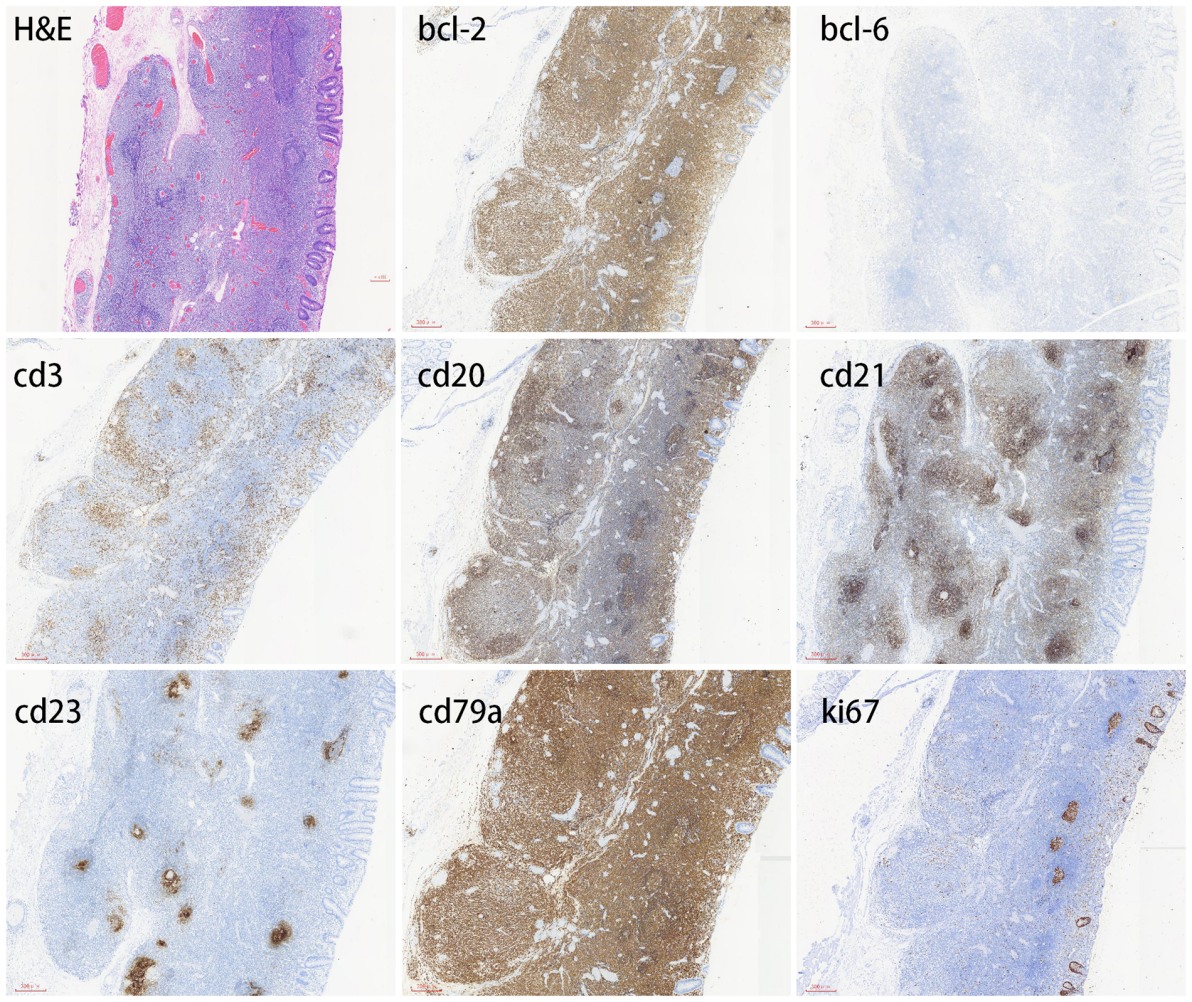
Immunohistochemical profile of colonic mucosa–associated lymphoid tissue lymphoma. MALT lymphoma is positive for CD20 and CD79a but negative for CD3 and Bcl-6. CD21 lymphocytes and FDC are positive, and CD23 FDC is also positive. Bcl-2 is usually positive. The Ki-67 proliferation index is typically low.

Herein, we describe a case of colonic MALT lymphoma in a 55-year-old man. The patient showed no clinical symptoms. He was routinely examined at Jinhua People's Hospital on 23 June 2021. The electronic gastroscopy suggested chronic gastritis, and the electronic colonoscopy revealed a mucosal redness in the sigmoid colon under white light (Figure 1A). A 0-IIb mucosal lesion of approximately 4.5 cm*5.0 cm with a red surface and clear borders was seen after indigo carmine staining (Figure 1B). The narrow-band imaging magnifying (NBI-M) endoscopy detected some branched abnormal blood vessels and the disappearance of the glandular structure (Figures 1C,D). In order to further define the nature of the lesion, the patient was hospitalized and underwent diagnostic endoscopic submucosal dissection (ESD). Preoperative routine blood tests, routine biochemical tests, hepatitis B virus, hepatitis C virus (HCV), human immunodeficiency virus (HIV), and syphilis tests showed no significant abnormalities. After full communication with the patient and obtaining his informed consent, diagnostic endoscopic submucosal dissection (ESD) was performed on him on 02 July 2021 (Figure 1E). Postoperative pathology suggested that the sigmoid colon was consistent with B-cell non-Hodgkin's lymphoma, with focal hyperplastic polyp formation in the extra-nodal marginal zone of MALT. Immunohistochemistry showed CD20, CD79a, and BCL2 (+), CD21 lymphocytes and FDC (+), CD23FDC (+), P53 partial (+), CD3, CD5, CD10, BCL6, and CyclinD1 (−), Ki67 (+, extra follicular 5%), and EBER (−) (Figure 2).

**Figure 3:**
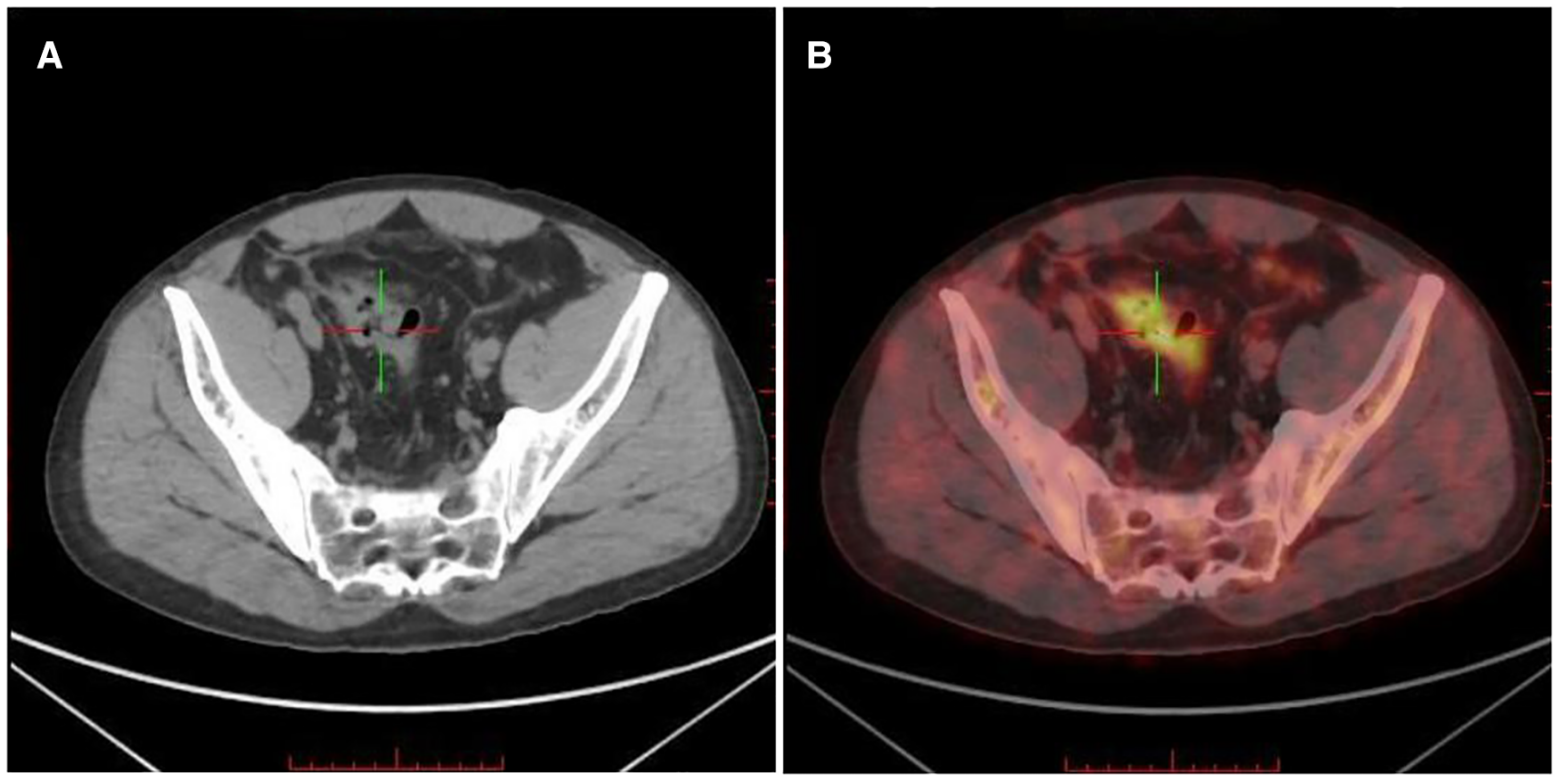
Positron emission computed tomography (PET-CT) of the lesion. (**A**) PET-CT showed a thickening of the sigmoid canal wall; (**B**) increased FDG uptake and a maximum SUV of approximately 7.8.

To further evaluate the staging of lymphoma and systemic metastasis, bone marrow aspiration and positron emission computed tomography (PET-CT) were performed on 15 July 2021. PET-CT showed a thickening of the sigmoid canal wall (Figure 3A), increased glucose metabolism, and a maximum SUV of approximately 7.8 (Figure 3B). The bone marrow aspiration report suggested no invasion of the bone marrow. Based on the PET-CT results suggesting increased glucose metabolism in the sigmoid colon, two possibilities were considered. First, it may be a false-positive due to the inflammatory response after colon ESD. Second, it is possible that the lymphoma may persist after colon ESD. After the patient was fully communicated and informed about his condition, he opted to undergo additional surgical treatment with partial colectomy and clearance of the peri-intestinal lymph nodes. Postoperative pathology showed no residual tumor in the original surgical field, negative surgical margins, and reactive pericolonic lymph nodes. The patient recovered well after surgery.

On 17 January 2022, the chest CT was repeated, which showed no significant abnormality. The abdominal CT examination suggested postoperative changes in the sigmoid colon, and no significant metastases were found. A repeat colonoscopy 1 year after surgery showed postsurgical scar formation and no recurrent lesions (Figure 1F).

## Discussion

Most non-Hodgkin's lymphoma (NHL) patients have superficial lymph node enlargement as the first symptom, and some patients have primary extranodal lymphoid tissue or organs. The MALT type of lymphoma in the extralymph node marginal zone can be found throughout the body, with the gastrointestine being the most common site of involvement, accounting for approximately 50% of all MALT lymphomas, followed by the parotid gland, skin, conjunctiva, head and neck, lung, thyroid, and breast (3, 4). In contrast, colorectal MALT lymphomas account for only 1.6% of all MALT lymphomas (5), and colorectal lymphomas account for only 0.2% of all colorectal malignancies with an annual incidence of 1.6/1 million (6). These results suggest a low incidence of colorectal MALT lymphoma. Bone marrow invasion is present in approximately 15%–20% of patients. About one-third of MALT lymphomas present as disseminated, and most MALT lymphomas are limited (7).

The etiology of MALT is unclear and may be related to persistent immune stimulation due to chronic infection or inflammation. Gastric MALT lymphoma is associated with chronic infection with HP, and a proportion of patients can be in remission after anti-HP treatment. Whether colonic MALT is associated with HP is inconclusive (8), which may be related to the small number of cases. Moreover, 22%–35% of non-gastric MALT lymphomas have HCV infection (9). Furthermore, chromosomal alterations t(11;18), t(1;14), t(14;18) are more common in MALT (10).

Relatively few cases of primary colonic MALT lymphoma have been reported in the literature, and common clinical manifestations are gastrointestinal bleeding, abdominal pain, perforation, and intussusception (11–13). The patient reported in this case was asymptomatic, and MALT was found during colonoscopy. The morphological changes of colonic MALT are diverse and can be single or multiple (14–16). Endoscopically, flat, elevated, polyp-like, or semipedunculated changes may be observed, and the surface may be smooth, granular, nodular, or ulcerative. The size can be up to 4–5 cm in a single lesion or in the form of multiple centimeter or subcentimeter nodules or polyps (17–20). If the endoscopic presentation of the lesion is atypical and susceptible to missed diagnosis or misdiagnosis, electronic staining endoscopy, combined with magnifying endoscopy, can improve the detection rate. Histologically, MALT lymphoma is a low-grade B-cell lymphoma that lacks pathomorphological and immunophenotypic features, and the small, shallow tissue of routine biopsies can cause diagnostic difficulties in pathology (21, 22). Diagnostic ESD procedures allow whole lesions to be excised and improve the diagnostic rate.

There is no standardized treatment protocol for colorectal MALT lymphoma. Treatment modalities include surgical resection, chemotherapy, radiotherapy, and endoscopic resection (14, 18, 23, 16). Rituximab is currently considered a treatment option for non-gastric MALT lymphoma, with an efficiency of approximately 80% (24, 25). ISRT also has good efficacy (26, 27). For extracolonic MALT lymphoma in certain specific sites such as the thyroid, lung, small intestine, and colon, surgical resection is an option. If the margins are positive, local regional ISRT should be administered after surgery, while follow-up observation can be an option for negative margins.

## Data Availability

The original contributions presented in the study are included in the article/Supplementary Material, and further inquiries can be directed to the corresponding author.
